# Facile synthesis of benzothiadiazine 1,1-dioxides, a precursor of RSV inhibitors, by tandem amidation/intramolecular aza-Wittig reaction

**DOI:** 10.3762/bjoc.9.54

**Published:** 2013-03-08

**Authors:** Krishna C Majumdar, Sintu Ganai

**Affiliations:** 1 Department of Chemistry, University of Kalyani, Kalyani 741235, W.B India

**Keywords:** benzothiadiazine-3-one 1,1-dioxide, 3-ethoxy-1,2,4-benzothiadiazine 1,1-dioxide, intramolecular aza-Wittig reaction, sultam

## Abstract

Reaction of *o*-azidobenzenesulfonamides with ethyl carbonochloridate afforded the corresponding amide derivatives, which gave 3-ethoxy-1,2,4-benzothiadiazine 1,1-dioxides through an intramolecular aza-Wittig reaction. The reaction was found to be general through the synthesis of a number of benzothiadiazine 1,1-dioxides. Acid-catalyzed hydrolysis of 3-ethoxy-1,2,4-benzothiadiazine 1,1-dioxides furnished the 2-substituted benzothiadiazine-3-one 1,1-dioxides in good yields and high purity, which is the core moiety of RSV inhibitors.

## Introduction

**Figure 1:**
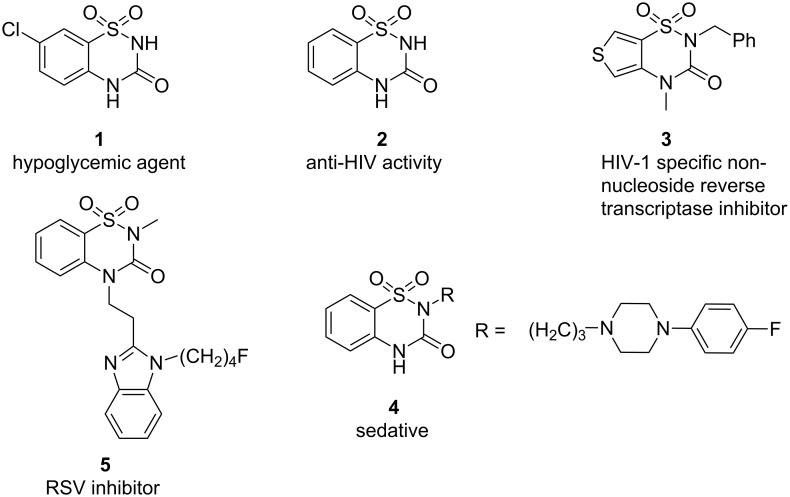
Biologically active 1,2,4-benzothiadiazine 1,1-dioxide derivatives.

Sultams have gained popularity in the scientific community especially among synthetic and medicinal chemists, because this basic moiety is present in many natural products and biologically active substances [1–8]. Especially, benzothiadiazine-3-one 1,1-dioxide and its derivatives possess potential activity, including hypoglycemic [9], anticancer and anti-HIV activity [10–13], and also serve as selective antagonists of CXR_2_ [14]. 2-Substituted-2*H*-1,2,4-benzothiadiazine-3(4*H*)one 1,1-dioxides showed varying degrees of sedative and hypotensive activities [15]. A number of benzothiadiazine 1,1-dioxide derivatives have recently been reported that display potent activity [16–22], including hypoglycemic (**1**), anti-HIV (**2**), HIV-1 specific non-nucleoside reverse transcriptase inhibitor (**3**), sedative (**4**), and respiratory syncytial virus (RSV) inhibitory activity (**5**; Figure 1).

A literature search revealed that the 1,2,4-benzothiadiazine 1,1-dioxides are generally synthesized either by condensation of *o*-aminobenzenesulfonamides with urea at elevated temperature [23] or by the reaction of *o*-aminobenzenesulfonamide with isocyanates in DMF under reflux [24]. Although various approaches to the preparation of 1,2,4-benzothiadiazine 1,1-dioxide derivatives have been reported [25–32], the development of a simpler method for the synthesis of the 1,2,4-benzothiadiazine 1,1-dioxide moiety is still desirable because of their biological significance.

The aza-Wittig reaction is employed for the construction of C=N, N=N and S=N double bonds in various heterocycles and heterocycle-containing natural products [33–43]. Recently, we have synthesized asymmetrically substituted piperazine-2,5-dione derivatives using the intramolecular aza-Wittig reaction [44]. In continuation of our earlier work [45–51], we have undertaken a study to synthesize 1,2,4-benzothiadiazine 1,1-dioxide derivatives using an intramolecular aza-Wittig reaction as the key step. Herein we report our results.

**Scheme 1:**
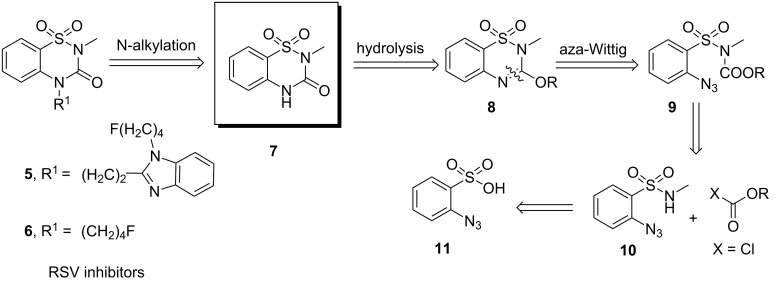
Retrosynthesis analysis of RSV inhibitors.

Retrosynthetic analysis of the RSV inhibitors **5** and **6** relied on benzothiadiazine-3-one 1,1-dioxide **7**, which can easily be obtained by simple hydrolysis of the benzothiadiazine 1,1-dioxide derivative **8**. Construction of this six-membered sultam **8** was thought to be achieved by intramolecular aza-Wittig reaction of the *o*-azido derivative **9**. The following retrosynthetic analysis led us to the starting material *o*-azidobenzenesulfonic acid (**11**) for the synthesis of the intermediate **10** necessary for the synthesis of RSV inhibitors (Scheme 1).

## Results and Discussion

**Scheme 2:**
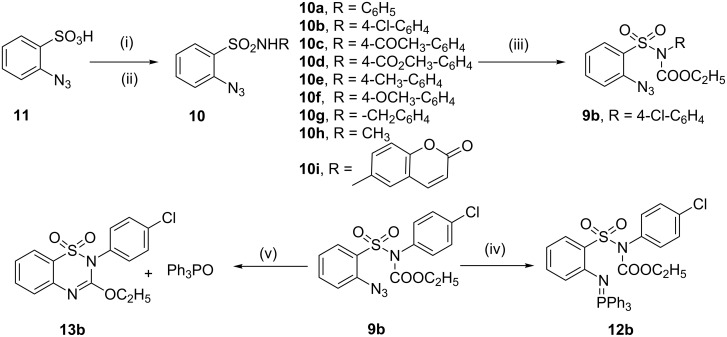
Preparation of 3-ethoxy-1,2,4-benzothiadiazine 1,1-dioxide. Reagent and conditions: (i) (COCl)_2_, DMF, CH_2_Cl_2_, reflux, 3 h; (ii) RNH_2_, NaOAc, MeOH + water, 60 °C; (iii) ClCO_2_C_2_H_5_, acetone, Et_3_N, rt, 5 h; (iv) PPh_3_, THF, reflux, 10 h; (v) PPh_3_, DCB, 135 °C, 8 h.

Sulfonic acid **11** bearing an *o*-azido group [30] was converted into the corresponding sulfonyl chloride by treatment with oxalyl chloride followed by the reaction with appropriate amines to give the requisite 2-azido-*N*-substituted benzenesulfonamides **10a–i**. The sulfonamide **10b** was reacted with ethyl carbonochloridate to afford the corresponding amide derivative **9b** required for our study. Initially, we turned our attention to the synthesis of a benzothiadiazine 1,1-dioxide derivative using substrate **9b** by intramolecular aza-Wittig reaction. To test this premise, **9b** was treated with triphenylphosphine in THF at room temperature, but no desired product was obtained, and only the intermediate iminophosphorane **12b** was isolated, even under reflux (Scheme 2).

We next conducted a series of reactions with the replacement of the solvent THF by other solvents, such as toluene, CH_2_Cl_2_, and CH_3_CN, but none of them afforded any cyclized product (Table 1, entries 2–4,). Then the reaction conditions were modified through the use of a higher-boiling-point solvent, i.e., *o*-dichlorobenzene (DCB). The reaction was successful at higher temperature, affording the desired cyclized product **13b** (54%) along with the by-product triphenylphosphine oxide (Table 1, entry 5).

**Table 1:** Summary of the intramolecular aza-Wittig reactions*.*^a^

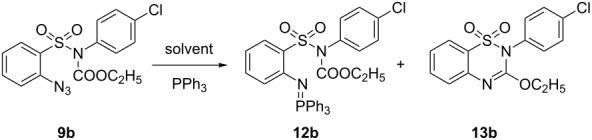				

Entry	Solvent	Temp (°C)	Time (h)	Yield (%)^b^

1^c^	THF	reflux	6	0
2^c^	toluene	120 °C	8	0
3^c^	CH_2_Cl_2_	reflux	8	0
4^c^	CH_3_CN	reflux	6	0
5	DCB	135 °C	8	54

^a^All the reactions were carried out with 1 equiv **9b** and 1.5 equiv PPh_3_; ^b^isolated yields of **13b**; ^c^only **12b** was separated.

Subsequently, we turned our attention to develop a simpler one-step procedure by heating the sulfonamide **10b** with ethyl carbonochloridate, Et_3_N and PPh_3_ in DCB at 135 °C for 6 h, which gave the cyclized product **13b** in 78% yield (Table 2, entry 1). The base Et_3_N was then replaced by Cs_2_CO_3_ or K_2_CO_3_, but no better result was obtained (Table 2, entries 2 and 3). Only DIPEA gave 69% yield of the product (Table 2, entry 4). However, surprisingly the use of xylene as the solvent improved the yield of the cyclized product (Table 2, entry 5). The replacement of NEt_3_ by DIPEA as the base also gave a similar yield of the product (Table 2, entry 6). The decomposition of the iminophosphorane intermediate into the corresponding amine derivative **14b** was found to occur at higher temperature (150 °C) producing a low yield of the cyclized product (Table 2, entry 7). The reaction did not occur at all in the absence of a base (Table 2, entry 8). The observations are summarized in Table 2.

**Table 2:** Summary of the intramolecular aza-Wittig reactions in a one-pot fashion.^a^

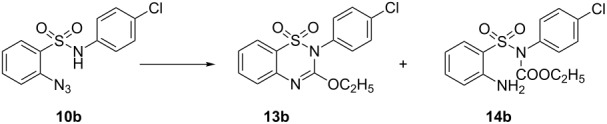					

Entry	Solvent	Base	Temp (°C)	Time (h)	Yield (%)^b^

1	DCB	Et_3_N	135 °C	6	78
2	DCB	K_2_CO_3_	135 °C	8	<30
3	DCB	Cs_2_CO_3_	135 °C	8	46
4	DCB	DIPEA	135 °C	6	69
**5**	**xylene**	**Et_3_N**	**135 °C**	**6**	**94**
6	xylene	DIPEA	135 °C	6	92
7^c^	xylene	Et_3_N	150 °C	6	5
8	xylene	–	135 °C	10	0

^a^All the reactions were carried out with 1 equiv **10b**, 1.5 equiv ClCO_2_Et, 2 equiv base, and 1.5 equiv PPh_3_; ^b^isolated yields of **13b**; ^c^a smaller amount of **13b** was isolated than the major product **14b**.

It is notable that xylene appears to be a suitable solvent for this reaction. We then carried out the reactions with a variety of substrates **10a–i** under the optimized conditions (ethyl carbonochloridate, PPh_3_, Et_3_N, xylene at 135 °C) in order to generalize the method, and the results are summarized in Table 3. The reactions of all the substrates having electron-deficient R-substituents at the 2-position proceeded smoothly, providing excellent yields, whereas the substrates having electron-donating R-substituents gave lower yields.

**Table 3:** Generalization of intramolecular aza-Wittig reaction.^a^

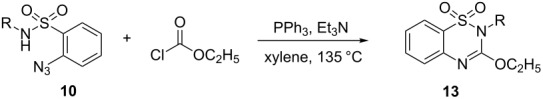				

Entry	*o*-azidosulfonamide	Time (h)	Product	Yield (%)^b^

1	**10a**, R = C_6_H_5_	8	**13a**, R = C_6_H_5_	90
2	**10b**, R = 4-Cl-C_6_H_4_	6	**13b**, R = 4-Cl-C_6_H_4_	94
3	**10c**, R = 4-COCH_3_-C_6_H_4_	6	**13c**, R = 4-COCH_3_-C_6_H_4_	92
4	**10d**, R = 4-CO_2_CH_3_-C_6_H_4_	6	**13d**, R = 4-CO_2_CH_3_-C_6_H_4_	95
5	**10e**, R = 4-CH_3_-C_6_H_4_	7	**13e**, R = 4-CH_3_-C_6_H_4_	80
6	**10f**, R = 4-OCH_3_-C_6_H_4_	7	**13f**, R = 4-OCH_3_-C_6_H_4_	83
7	**10g**, R = -CH_2_C_6_H_5_	7	**13g**, R = -CH_2_C_6_H_5_	87
8	**10h**, R = CH_3_	7	**13h**, R = CH_3_	79
9	**10i**, R = 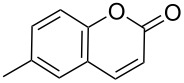	6	**13i**, R = 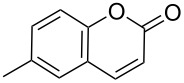	89

^a^Reaction conditions: Compound **10** (1 mmol), ClCO_2_C_2_H_5_ (1.5 mmol), Et_3_N (2 mmol) and PPh_3_ (1.5 mmol) were heated at 135 °C in xylene; ^b^isolated yields of compound **13**.

**Scheme 3:**
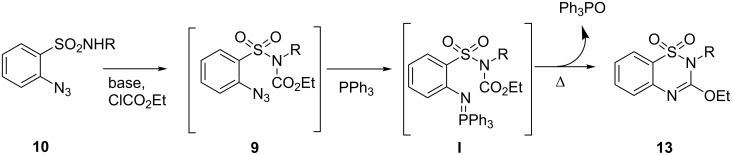
Rationalization of the formation of compound **13**.

The proposed mechanism for the formation of the products **13** may involve amidation of SO_2_NH_2_ by the reaction of nucleophilic sulfonamide **10** with ethyl carbonochloridate in the presence of Et_3_N to form the intermediates **9**, which may then undergo intramolecular aza-Wittig reaction via the formation of iminophosphorane intermediate **I**. We isolated iminophosphorane intermediate **12b** from the reaction with **10b** at room temperature. In the presence of heat the iminophosphorane intermediate **I** leads to the formation of the product 3-ethoxy-1,2,4-benzothiadiazine 1,1-dioxide **13** (Scheme 3). However, in all other cases we did not carry out the reactions at room temperature for isolation of the intermediates.

**Scheme 4:**
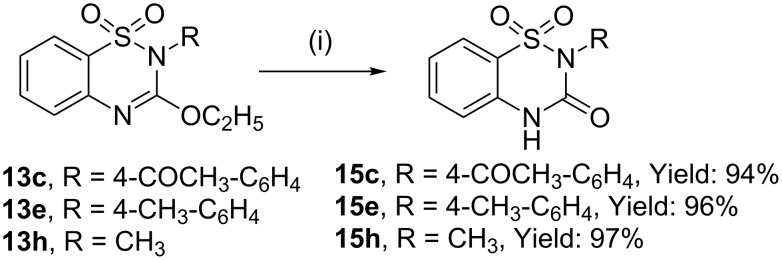
Preparation of benzothiadiazine-3-one 1,1-dioxide derivatives by acid-catalyzed hydrolysis; reagents and conditions: 50 mg of compound **13**, 1 mL HCl, 4 mL ethanol, 80 °C, 4 h.

We have also demonstrated the conversion of the products **13** to the 2-substituted benzothiadiazine-3-one 1,1-dioxide **15** by hydrolysing **13** with ethanolic HCl. The benzothiadiazine-3-one 1,1-dioxide derivatives **15c,e,h** were obtained in excellent yields from the compounds **13c,e,h** (Scheme 4). These 2-substituted benzothiadiazine-3-one 1,1-dioxides may further be alkylated at the 4-position with suitable halides to yield the RSV inhibitors **5** and **6** by using the reported [13] procedure.

Previously, Jung and Khazi [52] reported the synthesis of the benzothiadiazine 1,1-dioxide moiety from the reaction of *o*-aminobenzenesulfonamide with the costlier triphosgene, whereas in our case the synthesis of benzothiadiazine 1,1-dioxide derivatives was achieved from *o*-azidobenzenesulfonamides and required cheaper ethyl carbonochloridate as the reagent.

## Conclusion

In conclusion, we have developed a simple and efficient method for the synthesis of 3-ethoxybenzothiadiazine 1,1-dioxide and benzothiadiazine-3-one 1,1-dioxide derivatives starting from an easy precursor, by the application of an intramolecular aza-Wittig reaction. The reaction procedure is very simple and gives good to excellent yields of the products. This benzothiadiazine-3-one 1,1-dioxide can further be alkylated at the 4-position, following a literature procedure, to give the bioactive RSV inhibitors.

## Supporting Information

File 1 Experimental part.
