# Supplementary material for: Facile synthesis of benzothiadiazine 1,1-dioxides, a precursor of RSV inhibitors, by tandem amidation/intramolecular aza-Wittig reaction
Source: Beilstein J Org Chem. 2013 Mar 8;9:503–9. doi: 10.3762/bjoc.9.54 (PMC3629032; doi:10.3762/bjoc.9.54)
Supplement: File 1 — Experimental part. [file Beilstein_J_Org_Chem-09-503-s001.pdf]

Supporting Information

for

Facile synthesis of benzothiadiazine-1,1-dioxides, a precursor of RSV inhibitors, by tandem amidation/ intramolecular Aza-Wittig reaction

Krishna C. Majumdar*,[§] and Sintu Ganai

Address: Department of Chemistry, University of Kalyani, Kalyani 741235, W.B India

[§]Tel.: +91-33-2582 8750; Fax: +91-33-2582 8282

Email: Krishna C. Majumdar - kcm@klyuniv.ac.in

*Corresponding author

Experimental part

General information

Melting points were determined in open capillaries and are uncorrected. Silica gel (60–120 mesh) was used for chromatographic separation. Silica gel G was used for TLC. Petroleum ether (Pet) refers to the fraction boiling between 60 °C and 80 °C. IR spectra were recorded on a Perkin-Elmer L 120-000A spectrometer (ν_{max} in cm^{-1}) on KBr disks and neat. ^1H NMR and ^{13}C spectra were recorded in $CDCl_3$ and DMSO (chemical shifts in δ) with TMS as internal standard. MS were recorded on a Q-TOF micro™ instrument at the Indian

Institute of Chemical Biology (Kolkata). CHN analyses were recorded on a Perkin-Elmer 2400 series II CHN analyzer. HRMS were recorded on a Q-TOF Micro YA263 instrument at the Indian Association for the Cultivation of Science (Kolkata).

General procedure for the preparation of *o*-azidobenzenesulfonic acid (11) [1]**:**

2-Aminobenzenesulfonic acid (11.57 g, 66.8 mmol) was dissolved in a water (45 mL) and conc. H_2SO_4 (15 mL) mixture and cooled to 0 °C. A solution of $NaNO_2$ (6 g, 86.8 mmol) in water (30 mL) was added dropwise to it and the reaction mixture was stirred at 0 °C for 30 min. Then a cold solution of NaN_3 (8.68 g, 133.6 mmol) in water (30 mL) was added slowly. The reaction mixture was stirred at room temperature for a period of 12 h. The resulting precipitate was filtered off and washed with ice-cold water (10 mL) and dried under reduced pressure to give *o*-azidobenzenesulfonic acid (6.8 g, 34.1 mmol, 51%). Mp 152–154 °C (lit. 153–155 °C); IR (KBr): 1108, 1270, 1465, 1609, 2135 cm^{-1} (lit. 2134 cm^{-1}).

General procedure for the preparation of 2-azido-*N*-phenylbenzenesulfonamide (10a):

To a suspension of *o*-azidobenzenesulfonic acid (500 mg, 2.510 mmol) in dichloromethane (30 mL) was added a solution of 2 M oxalyl chloride in CH_2Cl_2 (6.280 mmol) and DMF (4 drop). The resulting mixture was heated under reflux for 3 h and then evaporated under reduced pressure. This crude *o*-azidobenzenesulfonyl chloride was added in small portions to a vigorously stirred mixture of the corresponding amine (here aniline 350 mg, 3.765 mmol) and NaOAc (310 mg, 3.765 mmol) in 50% aq MeOH (15 mL) over a period of 30 min. The mixture was then stirred at 60 °C for 1 h, cooled to room temperature, diluted with water (30 mL) and acidified to pH 2 with conc. HCl. The precipitate was filtered off, washed thoroughly with water and recrystallized from EtOAc to afford the grey solid product **10a** (571 mg, 2.084 mmol, 83%) [Found: C, 52.71; H, 3.86; N, 20.18 %. $C_{12}H_{10}N_4O_2S$ requires C, 52.54; H, 3.67; N, 20.43 %] R_f (15% EtOAc/Pet) 0.48; Mp 255–257 °C; IR (KBr): 1155, 1472, 2130, 3254 cm^{-1}; ^1H NMR (400 MHz, $CDCl_3$) δ_H 7.05-7.12 (m, 4H, ArH), 7.16-7.24 (m, 3H, ArH), 7.28 (s, 1H, NH), 7.53 (t, *J* = 8.0 Hz, 1H, ArH), 7.90 (d, *J* = 8.0 Hz, 1H, ArH); ^{13}C NMR

(100 MHz, $CDCl_3$) δ_C 119.4, 121.2, 124.8, 125.5, 128.9, 129.3, 131.3, 134.4, 136.1, 137.7; HRMS-ESI *m/z* found 275.0610 (MH^+), $C_{13}H_{10}N_4O_2S$ requires 275.0607 (MH^+).

Characterization of ethyl 2-azidophenylsulfonyl-(4-chlorophenyl)-carbamate (9b):

White gummy liquid; R_f (15% EtOAc/Pet) 0.58; IR (neat): 1158, 1332, 1510, 1711, 2134 cm^{-1}; ^1H NMR (400 MHz, $CDCl_3$) δ_H 1.08 (t, *J* = 7.2 Hz, 3H, $COOCH_2CH_3$), 4.09 (q, *J* = 7.2 Hz, 2H, $COOCH_2CH_3$), 7.32-7.36 (m, 2H, ArH), 7.40-7.46 (m, 4H, ArH), 7.68 (td, *J* = 6.4, 1.6 Hz, 1H, ArH), 8.22 (dd, *J* = 8.0, 1.6 Hz, 1H, ArH); ^{13}C NMR (100 MHz, $CDCl_3$) δ_C 13.9, 63.8, 119.8, 124.6, 129.3, 129.4, 131.5, 133.4, 133.5, 135.2, 135.4, 138.5, 151.9.

2-azido-*N*-(4-chlorophenyl)benzenesulfonamide (10b):

Using the general procedure (as **10a**) starting from *o*-azidobenzenesulfonic acid (500 mg, 2.510 mmol) and *p*-chloroaniline (480 mg, 3.765 mmol), compound **10b** was isolated as a gummy liquid (627 mg, 2.035 mmol, 81%); R_f (15% EtOAc/Pet) 0.51; IR (neat): 1160, 1331, 2136, 3251 cm^{-1}; ^1H NMR (400 MHz, $CDCl_3$) δ_H 7.04-7.07 (m, 3H, ArH), 7.17-7.19 (m, 2H, ArH), 7.22 (s, 1H, NH), 7.27 (d, *J* = 8.0 Hz, 1H, ArH), 7.56 (t, *J* = 7.6 Hz, 1H, ArH), 7.88 (d, *J* = 8.0 Hz, 1H, ArH); ^{13}C NMR (100 MHz, $CDCl_3$) δ_C 118.5, 121.8, 124.8, 125.4, 129.0, 129.1, 129.5, 132.5, 133.0, 136.5. 136.9; HRMS-ESI *m/z* found 331.0042 (MNa^+), $C_{12}H_9ClN_4O_2SNa$ requires 331.0032 (MNa^+).

Characterization of iminophosphorane intermediate (12b):

White solid; R_f (15% EtOAc/Pet) 0.45; Mp 128–130 °C; IR (KBr): 1158, 1332, 1548, 1711 cm^{-1}; ^1H NMR (400 MHz, $CDCl_3$) δ_H 1.12 (t, *J* = 6.8 Hz, 3H, $COOCH_2CH_3$), 4.03-4.15 (m, 2H, $COOCH_2CH_3$), 6.41 (d, *J* = 8.0 Hz, 1H, ArH), 6.66 (t, *J* = 7.6 Hz, 1H, ArH), 6.70-6.78 (m, 1H, ArH), 6.81 (d, *J* = 8.4 Hz, 1H, ArH), 6.99 (t, *J* = 7.6 Hz, 1H, ArH), 7.14 (d, *J* = 8.0 Hz, 1H, ArH), 7.33-7.38 (m, 7H, ArH), 7.47-7.54 (m, 3H, ArH), 7.63-7.68 (m, 5H, ArH), 7.72 (d, *J* = 8.0 Hz, 1H, ArH), 8.08 (d, *J* = 7.6 Hz, 1H, ArH); ^{13}C NMR (100 MHz, $CDCl_3$) δ_C 14.0, 63.7, 115.4, 116.9, 117.7, 119.4, 122.8, 122.9, 128.1, 128.7, 128.8, 129.0, 129.4, 130.0,

131.4, 131.7, 132.0, 132.1, 132.7, 132.8, 133.8, 134.1, 134.5, 134.9, 135.3, 135.4, 146.9, 151.7, 152.3, 152.5.

Characterization of ethyl 2-aminophenylsulfonyl-(4-chlorophenyl)-carbamate (14b):

White solid; R_f (15% EtOAc/Pet) 0.55; Mp 113–115 °C; IR (KBr): 1161, 1336, 1561, 1719, 3382, 3390 cm^{-1}; ^{1}H NMR (400 MHz, $CDCl_3$) δ_H 1.13 (t, J = 7.2 Hz, 3H, $COOCH_2CH_3$), 4.13 (q, J = 7.2 Hz, 2H, $COOCH_2CH_3$), 5.08 (m, 2H, NH_2), 6.72-6.79 (m, 2H, ArH), 7.28 (d, J = 8.8 Hz, 2H, ArH), 7.33-7.37 (m, 1H, ArH), 7.40 (d, J = 8.8 Hz, 2H, ArH), 7.72 (d, J = 8.0 Hz, 1H, ArH); ^{13}C NMR (100 MHz, $CDCl_3$) δ_C 13.8, 63.7, 119.7, 124.4, 128.9, 129.3, 131.5, 133.4, 133.5, 135.2, 135.4, 138.3, 151.4.

***N*-(4-acetylphenyl)-2-azidobenzenesulfonamide (10c):**

Using the general procedure (as **10a**) starting from *o*-azidobenzenesulfonic acid (500 mg, 2.510 mmol) and 1-(4-aminophenyl)ethanone (508 mg, 3.765 mmol), compound **10c** was isolated as a grey solid (690 mg, 2.183 mmol, 87%); [Found: C, 53.09; H, 3.88; N, 17.92; $C_{14}H_{12}N_4O_3S$ requires C, 53.16; H, 3.82; N, 17.71] R_f (15% EtOAc/Pet) 0.46; Mp decomposed at 200 °C; IR (KBr): 1160, 1339, 1669, 2102, 3208 cm^{-1}; ^{1}H NMR (400 MHz, $CDCl_3$) δ_H 2.51 (s, 3H, $COCH_3$), 7.19 (t, J = 8.2 Hz, 2H, ArH), 7.25-7,27 (m, 2H, ArH), 7.33 (s, 1H, NH), 7.58 (t, J = 7.8 Hz, 1H, ArH), 7.82 (d, J = 8.4 Hz, 2H, ArH), 7.99 (d, J = 7.6 Hz, 1H, ArH); ^{13}C NMR (100 MHz, $CDCl_3$) δ_C 26.4, 119.1, 119.6, 124.9, 129.9, 131.4, 133.6, 134.8, 137.8, 140.7, 196.6.

Methyl 4-(2-azidophenylsulfonamido)benzoate (10d):

Using the general procedure (as **10a**) starting from *o*-azidobenzenesulfonic acid (500 mg, 2.510 mmol) and methyl 4-aminobenzoate (569 mg, 3.765 mmol), compound **10d** was isolated as a grey solid (700 mg, 2.108 mmol, 84%); [Found: C, 50.71; H, 3.57; N, 16.93; $C_{14}H_{12}N_4O_4S$ requires C, 50.60; H, 3.64; N, 16.86] R_f (30% EtOAc/Pet) 0.47; Mp 188–190 °C; IR (KBr): 1165, 1345, 1695, 2106, 3212 cm^{-1}; ^{1}H NMR (400 MHz, DMSO) δ_H 3.75 (s, 3H, $COOCH_3$), 7.20 (d, J = 8.2 Hz, 2H, ArH), 7.31 (m, 1H, ArH), 7.49 (m, 1H, ArH), 7.64 (d, J =

7.6 Hz, 1H, ArH), 7.78 (d, *J* = 8.2 Hz, 2H, ArH), 7.95 (d, *J* = 7.0 Hz, 1H, ArH), 10.90 (s, 1H, NH); ^{13}C NMR (100 MHz, DMSO) δ_C 52.0, 117.9, 121.3, 124.3, 125.0, 128.6, 130.6, 130.8, 135.0, 137.7, 142.1, 165.7.

2-azido-*N*-*p*-tolylbenzenesulfonamide (10e):

Using the general procedure (as **10a**) starting from *o*-azidobenzenesulfonic acid (500 mg, 2.510 mmol) and *p*-methylaniline (403 mg, 3.765 mmol), compound **10e** was isolated as a white solid (636 mg, 2.208 mmol, 88%); [Found: C, 53.91; H, 4.12; N, 19.61 %. $C_{13}H_{12}N_4O_2S$ requires C, 54.15; H, 4.20; N, 19.43 %] R_f (15% EtOAc/Pet) 0.47; Mp 138–140 °C; IR (KBr): 1159, 1331, 2134, 3263 cm^{-1}; ^{1}H NMR (400 MHz, $CDCl_3$) δ_H 2.23 (s, 3H, CH_3), 6.98-7.02 (m, 5H, NH and ArH overlapped), 7.14 (dt, *J* = 8.4, 0.8 Hz, 1H, ArH), 7.26 (d, *J* = 8.8 Hz, 1H, ArH), 7.52 (dt, *J* = 8.4, 1.6 Hz, 1H, ArH), 7.86 (dd, *J* = 8.0, 1.2 Hz, 1H, ArH); ^{13}C NMR (100 MHz, $CDCl_3$) δ_C 20.8, 119.4, 121.9, 124.8, 129.0, 129.9, 131.3, 133.4, 134.3, 135.6, 137.6.

2-azido-*N*-(4-methoxyphenyl)benzenesulfonamide (10f):

Using the general procedure (as **10a**) starting from *o*-azidobenzenesulfonic acid (500 mg, 2.510 mmol) and *p*-methoxyaniline (463 mg, 3.765 mmol), compound **10f** was isolated as a grey solid (649 mg, 2.134 mmol, 85%); [Found: C, 51.03; H, 4.10; N, 18.57 %. $C_{13}H_{12}N_4O_3S$ requires C, 51.31; H, 3.97; N, 18.41 %] R_f (15% EtOAc/Pet) 0.48; Mp 116–118 °C; IR (KBr): 1163, 1311, 2139, 3277 cm^{-1}; ^{1}H NMR (400 MHz, $CDCl_3$) δ_H 3.72 (s, 3H, OCH_3), 6.72 (dd, *J* = 6.8, 2.0 Hz, 2H, ArH), 6.87 (s, 1H, NH), 7.01 (dd, *J* = 7.6, 2.0 Hz, 2H, ArH), 7.14 (t, *J* = 7.6 Hz, 1H, ArH), 7.29 (d, *J* = 8.0 Hz, 1H, ArH), 7.54 (dt, *J* = 8.0, 1.6 Hz, 1H, ArH), 7.80 (dd, *J* = 8.0, 1.6 Hz, 1H, ArH); ^{13}C NMR (100 MHz, $CDCl_3$) δ_C 55.4, 114.5, 119.3, 124.7, 124.8, 124.9, 128.6, 129.0, 131.3, 134.2, 137.6.

2-azido-*N*-benzylbenzenesulfonamide (10g):

Using the general procedure (as **10a**) starting from *o*-azidobenzenesulfonic acid (500 mg, 2.510 mmol) and benzylamine (403 mg, 3.675 mmol), compound **10g** was isolated as a light

tan solid (579 mg, 2.010 mmol, 80%); [Found: C, 53.81; H, 4.06; N, 19.65 %. $C_{13}H_{12}N_4O_2S$ requires C, 54.15; H, 4.20; N, 19.43 %] R_f (15% EtOAc/Pet) 0.50; Mp 80–82 °C; IR (KBr): 1165, 1327, 2142, 3292 cm^{-1}; ^1H NMR (400 MHz, $CDCl_3$) δ_H 4.10 (d, *J* = 6.0 Hz, 2H, NCH_2), 5.28 (brs, 1H, NH), 7.16-7.23 (m, 7H, ArH), 7.55 (t, *J* = 7.2 Hz, 1H, ArH), 7.98 (d, *J* = 7.6 Hz, 1H, ArH); ^{13}C NMR (100 MHz, $CDCl_3$) δ_C 47.6, 119.3, 124.8, 127.9, 128.0, 128.4, 128.5, 130.0, 130.6, 134.0, 136.0, 137.5.

2-azido-*N*-methylbenzenesulfonamide (10h):

Using the general procedure (as **10a**) starting from *o*-azidobenzenesulfonic acid (500 mg, 2.510 mmol) and methylamine solution (10 mL), compound **10h** was isolated as a colourless solid (442 mg, 2.084 mmol, 83%); [Found: C, 39.86; H, 4.05; N, 26.17 %. $C_7H_8N_4O_2S$ requires C, 39.62; H, 3.80; N, 26.40 %] R_f (15% EtOAc/Pet) 0.53; Mp 136–138 °C; IR (KBr): 1161, 1326, 2142, 3305 cm^{-1}; ^1H NMR (400 MHz, $CDCl_3$) δ_H 2.61 (d, *J* = 5.2 Hz, 3H, CH_3), 4.94 (d, *J* = 4.8 Hz, 1H, NH), 7.27 (d, *J* = 8.0 Hz, 1H, ArH), 7.31 (d, *J* = 8.4 Hz, 1H, ArH), 7.60 (dt, *J* = 8.8, 0.8 Hz, 1H, ArH), 7.99 (d, *J* = 7.6 Hz, 1H, ArH); ^{13}C NMR (100 MHz, $CDCl_3$) δ_C 29.4, 119.4, 124.9, 128.4, 131.1, 134.1, 137.6.

2-azido-*N*-(2-oxo-2*H*-chromen-6-yl)benzenesulfonamide (10i):

Using the general procedure (as **10a**) starting from *o*-azidobenzenesulfonic acid (500 mg, 2.510 mmol) and 6-aminocoumarine (605 mg, 3.765 mmol), compound **10i** was isolated as a brown solid (773 mg, 2.260 mmol, 90%); [Found: C, 52.78; H, 3.15; N, 16.11%. $C_{15}H_{10}N_4O_4S$ requires C, 52.63; H, 2.94; N, 16.37%.] R_f (30% EtOAc/Pet) 0.50; Mp 194–196 °C decomposed; IR (KBr):1160, 1310, 2128, 1711, 3282 cm^{-1}; ^1H NMR (400 MHz, DMSO) δ_H 6.43 (d, *J* = 9.6 Hz, 1H, =CH of coumarin), 7.26-7.35 (m, 3H, ArH), 7.43 (d, *J* = 2.4 Hz, 1H, ArH), 7.52 (d, *J* = 8.0 Hz, 1H, ArH), 7.64 (t, *J* = 7.6 Hz, 1H, ArH), 7.84 (d, *J* = 8.0 Hz, 1H, ArH), 8.00 (d, *J* = 9.6 Hz, 1H, =CH of coumarin), 10.48 (s, 1H, NH); ^{13}C NMR (100 MHz, $CDCl_3$ + DMSO) δ_C 116.9, 117.3, 118.7, 119.0, 119.8, 124.4, 124.6, 129.0, 131.0, 133.8, 134.3, 137.8, 143.2, 150.6, 160.4.

General procedure for the preparation of 2-*N*-phenyl-3-ethoxy-1,2,4-benzothiadiazine 1,1-dioxide (13a):

2-azido-*N*-phenylbenzenesulfonamide (150 mg, 0.547 mmol) and Et_3N (1.094 mmol) in 5 mL xylene was taken in a sealed tube and ethyl carbonochloridate (89 mg, 0.820 mmol) was added dropwise and stirred at room temperature for 30 minutes. Then Ph_3P (215 mg, 0.820 mmol) was added to it and it was sealed with the cap tightly and the reaction was heated at 135 °C. After completion of the reaction as monitored by TLC, the reaction mixture was cooled, water (20 mL) was added, and the mixture was extracted with ethyl acetate (3 × 30 mL). The ethyl acetate extract was washed with water (2 × 40 mL), followed by brine (30 mL). The organic layer was dried (over Na_2SO_4), and the solvent was evaporated to give a crude product. This was purified by column chromatography over silica gel (230–400 mesh) using petroleum ether and ethyl acetate (4:1) as an eluent. The product **13a** was isolated as a white solid (149 mg, 0.493 mmol, 90%); R_f (20% EtOAc/Pet) 0.47; Mp 116–118 °C; IR (KBr): 1155, 1306, 1338, 1579, 1610 cm^{-1}; ^1H NMR (300 MHz, $CDCl_3$) δ_H 1.26 (t, *J* = 6.9 Hz, 3H, OCH_2CH_3), 4.45 (q, *J* = 6.9 Hz, 2H, OCH_2CH_3), 7.33-7.56 (m, 7H, ArH), 7.66 (td, *J* = 7.5, 3.0 Hz, 1H, ArH), 7.89 (d, *J* = 7.2 Hz, 1H, ArH); ^{13}C NMR (100 MHz, $CDCl_3$) δ_C 14.0, 65.1, 122.0, 124.7, 126.1, 126.7, 129.3, 129.6, 129.8, 131.4, 133.8, 141.8, 150.7; HRMS-ESI *m/z* found 303.0801 (MH^+), $C_{15}H_{15}N_2O_3S$ requires 303.0803 (MH^+).

2-*N*-(4-chlorophenyl)-3-ethoxy-1,2,4-benzothiadiazine 1,1-dioxide (13b):

Using the general procedure (as **13a**) starting from 2-azido-*N*-(4-chlorophenyl)benzenesulfonamide (150 mg, 0.487 mmol), compound **13b** was isolated as a white solid (154 mg, 0.458 mmol, 94%); R_f (20% EtOAc/Pet) 0.55; Mp 102–104 °C; IR (KBr): 1186, 1310, 1338, 1577, 1610 cm^{-1}; ^1H NMR (300 MHz, $CDCl_3$) δ_H 1.25 (t, *J* = 7.2 Hz, 3H, OCH_2CH_3), 4.43 (q, *J* = 6.9 Hz, 2 , OCH_2CH_3), 7.34-7.38 (m, 3H, ArH), 7.44 (d, *J* = 8.4 Hz, 3H, ArH), 7.65 (t, *J* = 7.8 Hz, 1H, ArH), 7.86 (d, *J* = 8.0 Hz, 1H, ArH); ^{13}C NMR (100 MHz, $CDCl_3$) δ_C 14.0, 65.3, 122.0, 124.9, 125.9, 126.8, 129.6, 129.8, 131.1, 134.0, 135.8, 141.7,

150.3; HRMS-ESI *m*/*z* found 337.0409 (MH^+), 339.0380 (MH^++2), $C_{15}H_{14}ClN_2O_3S$ requires 337.0414 (MH^+), 339.0384 (MH^++2).

2-*N*-(4-acetylphenyl)-3-ethoxy-1,2,4-benzothiadiazine 1,1-dioxide (13c):

Using the general procedure (as **13a**) starting from *N*-(4-acetylphenyl)-2-azidobenzenesulfonamide (150 mg, 0.474 mmol), compound **13c** was isolated as a white solid (150 mg, 0.436 mmol, 92%); R_f (20% EtOAc/Pet) 0.48; Mp 143–145 °C; IR (KBr): 1159, 1311, 1585, 1615, 1682 cm^{-1}; ^{1}H NMR (400 MHz, $CDCl_3$) δ_H 1.25 (t, *J* = 6.8 Hz, 3H, OCH_2CH_3), 2.65 (s, 3H, $COCH_3$), 4.45 (q, *J* = 6.8 Hz, 2H, OCH_2CH_3), 7.36 (t, *J* = 7.6 Hz, 1H, ArH), 7.46-7.51 (m, 3H, ArH), 7.66 (t, *J* = 7.8 Hz, 1H, ArH); 7.86 (d, *J* = 8.0 Hz, 1H, ArH), 8.05 (d, *J* = 8.4 Hz, 2H, ArH); ^{13}C NMR (100 MHz, $CDCl_3$) δ_C 12.0, 26.7, 65.4, 122.0, 125.0, 126.0, 126.9, 129.3, 129.7, 134.0, 135.8, 137.7, 141.7, 151.1, 197.0; HRMS-ESI *m*/*z* found 345.0903 (MH^+), $C_{17}H_{17}N_2O_4S$ requires 345.0909 (MH^+).

2-*N*-(*p*-methoxycarbonylphenyl)-3-ethoxy-1,2,4-benzothiadiazine 1,1-dioxide (13d):

Using the general procedure (as **13a**) starting from methyl 4-(2-azidophenylsulfonamido)benzoate (150 mg, 0.451 mmol), compound **13d** was isolated as a white solid (154 mg, 0.429 mmol, 95%); R_f (20% EtOAc/Pet) 0.45; Mp 160–162 °C; IR (KBr): 1179, 1312, 1583, 1619, 1718 cm^{-1}; ^{1}H NMR (400 MHz, $CDCl_3$) δ_H 1.24 (t, *J* = 7.2 Hz, 3H, OCH_2CH_3), 3.95 (s, 3H, $COOCH_3$), 4.44 (q, *J* = 7.2 Hz, 2H, OCH_2CH_3), 7.36 (td, *J* = 7.6, 0.8 Hz, 1H, ArH), 7.45-7.48 (m, 3H, ArH), 7.65 (td, *J* = 8.4, 1.4 Hz, 1H, ArH), 7.86 (dd, *J* = 8.0, 1.2 Hz, 1H, ArH), 8.14 (dd, *J* = 8.4, 1.6 Hz, 2H, ArH); ^{13}C NMR (100 MHz, $CDCl_3$) δ_C 14.0, 52.4, 65.3, 122.0, 125.0, 126.1, 126.9, 129.6, 130.6, 131.2, 134.0, 135.7, 141.7, 150.2, 166.1; HRMS-ESI *m*/*z* found 361.0849 (MH^+), $C_{17}H_{17}N_2O_5S$ requires 361.0858 (MH^+).

2-*N*-*p*-tolyl-3-ethoxy-1,2,4-benzothiadiazine 1,1-dioxide (13e):

Using the general procedure (as **13a**) starting from 2-azido-*N*-*p*-tolylbenzenesulfonamide (150 mg, 0.520 mmol), compound **13e** was isolated as a white solid (132 mg, 0.417 mmol, 80%); R_f (20% EtOAc/Pet) 0.53; Mp 108–110 °C; IR (KBr): 1156, 1305, 1340, 1580,

1611 cm^{-1}; ^{1}H NMR (300 MHz, $CDCl_3$) δ_H 1.25 (t, *J* = 7.2 Hz, 3H, OCH_2CH_3), 2.41 (s, 3H, *p*-$ArCH_3$), 4.43 (q, *J* = 7.2 Hz, 2H, OCH_2CH_3), 7.25-7.32 (m, 4H, ArH), 7.33 (t, *J* = 7.6 Hz, 1H, ArH), 7.44 (d, *J* = 8.0 Hz, 1H, ArH), 7.62 (t, *J* = 7.6 Hz, 1H, ArH), 7.86 (d, *J* = 7.6 Hz, 1H, ArH); ^{13}C NMR (75 MHz, $CDCl_3$) δ_C 14.1, 21.3, 65.1, 122.0, 124.7, 126.1, 126.7, 128.6, 129.6, 130.1, 133.8, 139.9, 142.0, 150.9; HRMS-ESI *m*/*z* found 317.0965 (MH^+), $C_{16}H_{17}N_2O_3S$ requires 317.0960 (MH^+).

2-*N*-(4-methoxyphenyl)-3-ethoxy-1,2,4-benzothiadiazine 1,1-dioxide (13f):

Using the general procedure (as **13a**) starting from 2-azido-*N*-(4-methoxyphenyl)benzenesulfonamide (150 mg, 0.493 mmol), compound **13f** was isolated as a white solid (136 mg, 0.409 mmol, 83%); R_f (20% EtOAc/Pet) 0.50; Mp 148–150 °C; IR (KBr): 1167, 1334, 1581, 1611 cm^{-1}; ^{1}H NMR (300 MHz, $CDCl_3$) δ_H 1.27 (t, *J* = 6.9 Hz, 3H, OCH_2CH_3), 3.87 (s, 3H, OCH_3), 4.44 (q, *J* = 6.9 Hz, 2H, OCH_2CH_3), 6.98 (d, *J* = 8.7 Hz, 2H, ArH), 7.28-7.37 (m, 3H, ArH), 7.45 (d, *J* = 7.8 Hz, 1H, ArH), 7.63 (td, *J* = 8.4, 1.5 Hz, 1H, ArH), 7.88 (d, *J* = 7.8 Hz, 1H, ArH); ^{13}C NMR (100 MHz, $CDCl_3$) δ_C 14.0, 55.5, 65.0, 114.6, 122.1, 123.4, 124.6, 126.0, 126.7, 131.1, 133.8, 141.8, 150.9, 160.5; HRMS-ESI *m*/*z* found 333.0911 (MH^+), $C_{16}H_{17}N_2O_4S$ requires 333.0909 (MH^+).

2-*N*-benzyl-3-ethoxy-1,2,4-benzothiadiazine 1,1-dioxide (13g):

Using the general procedure (as **13a**) starting from 2-azido-*N*-benzylbenzenesulfonamide (150 mg, 0.520 mmol), compound **13g** was isolated as a white solid (143 mg, 0.452 mmol, 87%); R_f (20% EtOAc/Pet) 0.54; Mp 85–87 °C; IR (KBr): 1152, 1327, 1584, 1613 cm^{-1}; ^{1}H NMR (400 MHz, $CDCl_3$) δ_H 1.28 (t, *J* = 7.2 Hz, 3H, OCH_2CH_3), 4.40 (q, *J* = 7.2 Hz, 2H, OCH_2CH_3), 5.04 (s, 2H, NCH_2Ph), 7.25-7.36 (m, 7H, ArH), 7.57 (td, *J* = 8.0, 1.2 Hz, 1H, ArH), 7.86 (d, *J* = 8.0 Hz, 1H, ArH); ^{13}C NMR (100 MHz, $CDCl_3$) δ_C 14.0, 45.2, 65.1, 121.7, 124.6, 125.5, 126.6, 127.9, 128.3, 128.5, 133.7, 135.9, 141.7, 150.9; HRMS-ESI *m*/*z* found 317.0967 (MH^+), $C_{16}H_{17}N_2O_3S$ requires 317.0960 (MH^+).

2-*N*-methyl-3-ethoxy-1,2,4-benzothiadiazine 1,1-dioxide (13h):

Using the general procedure (as **13a**) starting from 2-azido-*N*-methylbenzenesulfonamide (150 mg, 0.707 mmol), compound **13h** was isolated as a white solid (134 mg, 0.558 mmol, 79%); R_f (20% EtOAc/Pet) 0.45; Mp 136–138 °C; IR (KBr): 1156, 1305, 1340, 1580, 1611 cm^{-1}; ^1H NMR (400 MHz, $CDCl_3$) δ_H 1.42 (t, *J* = 7.2 Hz, 3H, OCH_2CH_3), 3.38 (s, 3H, NCH_3), 4.49 (q, *J* = 7.2 Hz, 2H, OCH_2CH_3), 7.28 (t, *J* = 7.8 Hz, 1H, ArH), 7.35 (d, *J* = 8.0 Hz, 1H, ArH), 7.58 (td, *J* = 7.8, 1.2 Hz, 1H, ArH), 7.82 (d, *J* = 8.0 Hz, 1H, ArH); ^{13}C NMR (100 MHz, $CDCl_3$) δ_C 14.2, 27.1, 65.0, 121.6, 124.4, 124.7, 126.4, 133.7, 141.8, 151.3; HRMS-ESI *m*/*z* found 241.0639 (MH^+), $C_{10}H_{13}N_2O_3S$ requires 241.0647 (MH^+).

2-*N*-(2-oxo-2*H*-chromen-6-yl)-3-ethoxy-1,2,4-benzothiadiazine 1,1-dioxide (13i):

Using the general procedure (as **13a**) starting from 2-azido-*N*-(2-oxo-2*H*-chromen-6-yl)benzenesulfonamide (150 mg, 0.438 mmol), compound **13i** was isolated as a white solid (144 mg, 0.390 mmol, 89%); R_f (25% EtOAc/Pet) 0.40; Mp 193–195 °C; IR (KBr): 1186, 1339, 1592, 1629, 1733 cm^{-1}; ^1H NMR (300 MHz, $CDCl_3$) δ_H 1.27 (t, *J* = 6.9 Hz, 3H, OCH_2CH_3), 4.46 (q, *J* = 6.9 Hz, 2H, OCH_2CH_3), 6.51 (d, *J* = 9.6 Hz, 1H, =CH of coumarin), 7.35-7.55 (m, 4 H, ArH), 7.63-7.68 (m, 2H, ArH), 7.73 (d, *J* = 9.6 Hz, 1H, =CH of coumarin), 7.88 (d, *J* = 8.1 Hz, 1H, ArH); ^{13}C NMR (75 MHz, $CDCl_3$) δ_C 14.1, 65.5, 117.7, 118.1, 119.4, 122.1, 125.0, 125.8, 127.0, 127.3, 129.3, 133.2, 134.2, 141.6, 142.7, 154.5, 160.0; HRMS-ESI *m*/*z* found 371.0710 (MH^+), $C_{18}H_{15}N_2O_5S$ requires 371.0702 (MH^+).

General procedure for the preparation of 2-*N*-substituted-1,2,4-benzothiadiazine-3-one 1,1-dioxide derivatives (15):

50 mg 3-ethoxy-1,2,4-benzothiadiazine 1,1-dioxide (**13**) was taken in a round bottomed flask in ethanol (4 mL) and HCl (1 mL) was added to it. The reaction mixture was heated at 80 °C for 4 h. After completion of the reaction as monitored by TLC the solvent was evaporated and saturated $NaHCO_3$ solution (10 mL) was added to it and extracted with ethyl acetate (3 × 10 mL). The ethyl acetate extract was washed with water (2 × 15 mL), followed by brine

(10 mL). The organic layer was dried (over Na_2SO_4), and the solvent was evaporated and passed through a short column to yield solid product **15**.

2-*N*-(4-acetylphenyl)-1,2,4-benzothiadiazine-3-one 1,1-dioxide (15c):

Using the general procedure starting from compound **13c** (50 mg, 0.145 mmol), compound **15c** was isolated as a white solid (43 mg, 0.136 mmol, 94%); R_f (30% EtOAc/Pet) 0.42; Mp 123–125 °C; IR (KBr): 1181, 1340, 1608, 1686, 1698, 3076 cm^{-1}; ^1H NMR (400 MHz, $CDCl_3$ + DMSO) δ_H 2.49 (s, 3 H, $COCH_3$), 7.25-7.34 (m, 2H, ArH), 7.58-7.62 (m, 3H, ArH), 7.84 (d, *J* = 7.6 Hz, 1H, ArH), 8.08 (d, *J* = 8.0 Hz, 2H, ArH), 11.19 (s, 1H, NH); ^{13}C NMR (100 MHz, $CDCl_3$ + DMSO) δ_C 26.8, 117.7, 122.7, 123.0, 123.3, 129.4, 130.8, 134.6, 134.8, 135.1, 137.8, 150.1, 197.1; HRMS-ESI *m*/*z* found 317.0591 (MH^+), $C_{15}H_{13}N_2O_4S$ requires 317.0596 (MH^+).

2-*N*-*p*-tolyl-1,2,4-benzothiadiazine-3-one 1,1-dioxide (15e):

Using the general procedure starting from compound **13e** (50 mg, 0.158 mmol), compound **15e** was isolated as a white solid (44 mg, 0.152 mmol, 96%); R_f (30% EtOAc/Pet) 0.47; Mp 250–252 °C; IR (KBr): 1187, 1345, 1360, 1602, 1708, 3089 cm^{-1}; ^1H NMR (400 MHz, $CDCl_3$) δ_H 2.43 (s, 3H, *p*-$ArCH_3$), 7.01 (d, *J* = 8.0 Hz, 1H, ArH), 7.28-7.37 (m, 5H, ArH), 7.58 (td, *J* = 8.4, 1.0 Hz, 1H, ArH), 7.89 (d, *J* = 8.0 Hz, 1H, ArH), 9.01 (s, 1H, NH); ^{13}C NMR (100 MHz, $CDCl_3$) δ_C 21.4, 117.3, 123.1, 123.2, 123.8, 127.6, 130.2, 130.4, 134.0, 134.5, 140.4, 151.3; HRMS-ESI *m*/*z* found 289.0651 (MH^+), $C_{14}H_{13}N_2O_3S$ requires 289.0647 (MH^+).

2-*N*-methyl-1,2,4-benzothiadiazine-3-one 1,1-dioxide (15h):

Using the general procedure starting from compound **13h** (50 mg, 0.208 mmol), compound **15h** was isolated as a white solid (43 mg, 0.202 mmol, 97%); R_f (30% EtOAc/Pet) 0.53; Mp 158–160 °C; IR (KBr): 1180, 1333, 1600, 1693, 3061 cm^{-1}; ^1H NMR (400 MHz, $CDCl_3$) δ_H 3.42 (s, 3H, NCH_3), 7.18 (d, *J* = 8.0 Hz, 1H, ArH), 7.29 (t, *J* = 7.8 Hz, 1H, ArH), 7.61 (t, *J* = 7.8 Hz, 1H, ArH), 7.85 (d, *J* = 7.6 Hz, 1H, ArH), 10.34 (s, 1H, NH); ^{13}C NMR (100 MHz,

$CDCl_3$) δ_C 26.5, 117.1, 122.0, 122.8, 123.9, 134.0, 134.6, 152.0; HRMS-ESI *m*/*z* found 213.0331 (MH^+), $C_{15}H_{15}N_2O_3S$ requires 213.0334 (MH^+).

Reference

1. Blackburn, C.; Achab, A.; Elder, A.; Ghosh, S.; Guo, J.; Harriman, G.; Jones, M. *J. Org. Chem.* **2005**, *70*, 10206-10209. doi: 10.1021/jo051843h
